# Cerebrospinal fluid oligoclonal bands in Chinese patients with multiple sclerosis: the prevalence and its association with clinical features

**DOI:** 10.3389/fimmu.2023.1280020

**Published:** 2023-11-16

**Authors:** Xiang Zhang, Hongjun Hao, Tao Jin, Wei Qiu, Huan Yang, Qun Xue, Jian Yin, Ziyan Shi, Hai Yu, Xiaopei Ji, Xiaobo Sun, Qiuming Zeng, Xiaoni Liu, Jingguo Wang, Huining Li, Xiaoyan He, Jing Yang, Yarong Li, Shuangshuang Liu, Alexander Y. Lau, Feng Gao, Shimin Hu, Shuguang Chu, Ding Ding, Hongyu Zhou, Haifeng Li, Xiangjun Chen

**Affiliations:** ^1^ Department of Neurology, Huashan Hospital, Fudan University and Institute of Neurology, Fudan University, National Center for Neurological Disorders, Shanghai, China; ^2^ Department of Neurology, Peking University First Hospital, Beijing, China; ^3^ Department of Neurology and Neuroscience Center, The First Hospital of Jilin University, Changchun, China; ^4^ Department of Neurology, The Third Affiliated Hospital of Sun Yat-sen University, Guangzhou, China; ^5^ Department of Neurology, Xiangya Hospital, Central South University, Changsha, China; ^6^ Department of Neurology, The First Affiliated Hospital of Soochow University, Suzhou, China; ^7^ Department of Neurology, Beijing Hospital, Beijing, China; ^8^ Department of Neurology, West China Hospital, Sichuan University, Chengdu, China; ^9^ Department of Neurology, Tianjin Neurological Institute, Tianjin Medical University General Hospital, Tianjin, China; ^10^ Department of Neurology, The Xinjiang Uygur Autonomous Region People’s Hospital, Urumqi, China; ^11^ Department of Neurology, The First Affiliated Hospital of Zhengzhou University, Zhengzhou, China; ^12^ Department of Neurology, Xuanwu Hospital, Capital Medical University, Beijing, China; ^13^ Department of Medicine and Therapeutics, Prince of Wales Hospital, The Chinese University of Hong Kong, Hong Kong, Hong Kong SAR, China; ^14^ Department of Clinical Epidemiology and Evidence-Based Medicine, Xuanwu Hospital Capital Medical University, Beijing, China; ^15^ Department of Radiology, Shanghai East Hospital, Tongji University School of Medicine, Shanghai, China

**Keywords:** oligoclonal bands, cerebrospinal fluid, multiple sclerosis, prevalence, diagnostic performance, China

## Abstract

**Background:**

Cerebrospinal fluid oligoclonal band (CSF-OCB) is an established biomarker in diagnosing multiple sclerosis (MS), however, there are no nationwide data on CSF-OCB prevalence and its diagnostic performance in Chinese MS patients, especially in the virtue of common standard operation procedure (SOP).

**Methods:**

With a consensus SOP and the same isoelectric focusing system, we conducted a nationwide multi-center study on OCB status in consecutively, and recruited 483 MS patients and 880 non-MS patients, including neuro-inflammatory diseases (NID, n = 595) and non-inflammatory neurological diseases (NIND, n=285). Using a standardized case report form (CRF) to collect the clinical, radiological, immunological, and CSF data, we explored the association of CSF-OCB positivity with patient characters and the diagnostic performance of CSF-OCB in Chinese MS patients. Prospective source data collection, and retrospective data acquisition and statistical data analysis were used.

**Findings:**

369 (76.4%) MS patients were OCB-positive, while 109 NID patients (18.3%) and 6 NIND patients (2.1%) were OCB-positive, respectively. Time from symptom onset to diagnosis was significantly shorter in OCB-positive than that in OCB-negative MS patients (13.2 vs 23.7 months, P=0.020). The prevalence of CSF-OCB in Chinese MS patients was significantly higher in high-latitude regions (41°-50°N)(P=0.016), and at high altitudes (>1000m)(P=0.025). The diagnostic performance of CSF-OCB differentiating MS from non-MS patients yielded a sensitivity of 76%, a specificity of 87%.

**Interpretation:**

The nationwide prevalence of CSF-OCB was 76.4% in Chinese MS patients, and demonstrated a good diagnostic performance in differentiating MS from other CNS diseases. The CSF-OCB prevalence showed a correlation with high latitude and altitude in Chinese MS patients.

## Introduction

Multiple sclerosis (MS) is a typical chronic inflammatory demyelinating disease of the central nervous system (CNS) (1). The clinical manifestations of MS are diverse, and the core diagnostic points are neurological deficits disseminated in time and space. The diagnosis of MS is challenging, and it should be prudent to differentiate it from other diseases with similar clinical manifestations (2, 3), especially in other inflammatory demyelinating diseases, such as neuromyelitis optica spectrum disorders (NMOSD), and myelin oligodendrocyte glycoprotein antibody-associated disease (MOGAD). Therefore, MS-related biomarkers have become the focus of ongoing research. Although in recent years many diagnostic biomarkers have been reported to be related to MS, few of them have clinical applicability and reliability (4, 5). The presence of immunoglobulin G (IgG) oligoclonal band (OCB) in cerebrospinal fluid (CSF) indicates intrathecal synthesis of immunoglobulin in response to chronic inflammation in CNS (6, 7). CSF-OCB was found in MS patients in the 1960s (8). Since then, it was confirmed as an established biomarker in diagnosing MS and is widely used in the diagnosis of MS globally (9–14).

In the 2017 McDonald diagnostic criteria of MS, OCB is included and can be used as a substitution for dissemination in time (15), which promotes the early diagnosis of MS in patients with the clinically isolated syndrome (CIS) (13, 16, 17). However, the expert panel emphasized that this criteria should be used prudently in Asian patients (15), because of the higher prevalence of non-MS demyelinating diseases in Asia and some of these patients also have CSF-OCB but with short segmental spinal lesions and atypical cerebral lesions. Due to the more important role of CSF-OCB in the diagnosis of MS, Chinese scholars recognized the lack of nationwide data on CSF-OCB in Chinese MS patients might lead to under or over-diagnosis of MS. CSF-OCB was reported in over 85% of MS patients in Europe and the United States (14, 18, 19). However, the prevalence of CSF-OCB in Chinese MS patients was reported as about 30~70% by several single centers (17, 20–23), and was lower than that reported in Western countries. In China, MS was defined as a rare disease (24) with an estimated prevalence of 1~5 per 100,000 (25). Nevertheless, due to the large population in China, the total number of MS patients in China is still large. Till now there are no nationwide data on CSF-OCB positivity and its diagnostic performance in Chinese MS patients.

Using different testing methods of CSF-OCB in different regional studies was previously presumed as the difference in reported OCB positivity in Chinese MS patients and the difference from that reported in Western countries. China is a vast country, hence there might be differences in regional CSF-OCB prevalence due to differences in latitude and altitude. There are also different backgrounds in culture and conventions in different regions of China. Therefore, experts in 12 regional referring MS centers in mainland of China formed the Multiple Sclerosis Collaborative Research Group in 2019 and started the project “CNS-OCB, China National Study for Oligo-Clonal Band”in 2020. We first developed a consensus standard operation procedure (SOP) with an isoelectric focusing system and validated the inter-laboratory agreement (26). With this SOP and the same isoelectric focusing system, we conducted a nationwide multi-center study on OCB status in consecutively, and recruited 483 MS patients and 880 non-MS patients. Using a standardized case report form (CRF) to collect the clinical, radiological, immunological, and CSF data, we explored the association of CSF-OCB positivity with patient characters and the diagnostic performance of CSF-OCB in Chinese MS patients.

## Methods

### Patients

All patients included in this study were diagnosed with MS or non-MS diseases in the recruiting centers from May 2020 to May 2022 (ChiCTR2000040363). This study was approved by the Ethics Committee of Shanghai Huashan Hospital and all other participating centers. The study was conducted according to the principles of the Declaration of Helsinki. All patients have signed the informed consent and agreed with sample collection and data publication. Prospective source data collection, and retrospective data acquisition and statistical data analysis were used. The data was collected at all centers with a structured database issued by a cooperation project (“CNS-OCB, China National Study for Oligo-Clonal Band”) from 2019, and acquired from the database of each center according to the inclusion and exclusion criteria.

**Figure 1:**
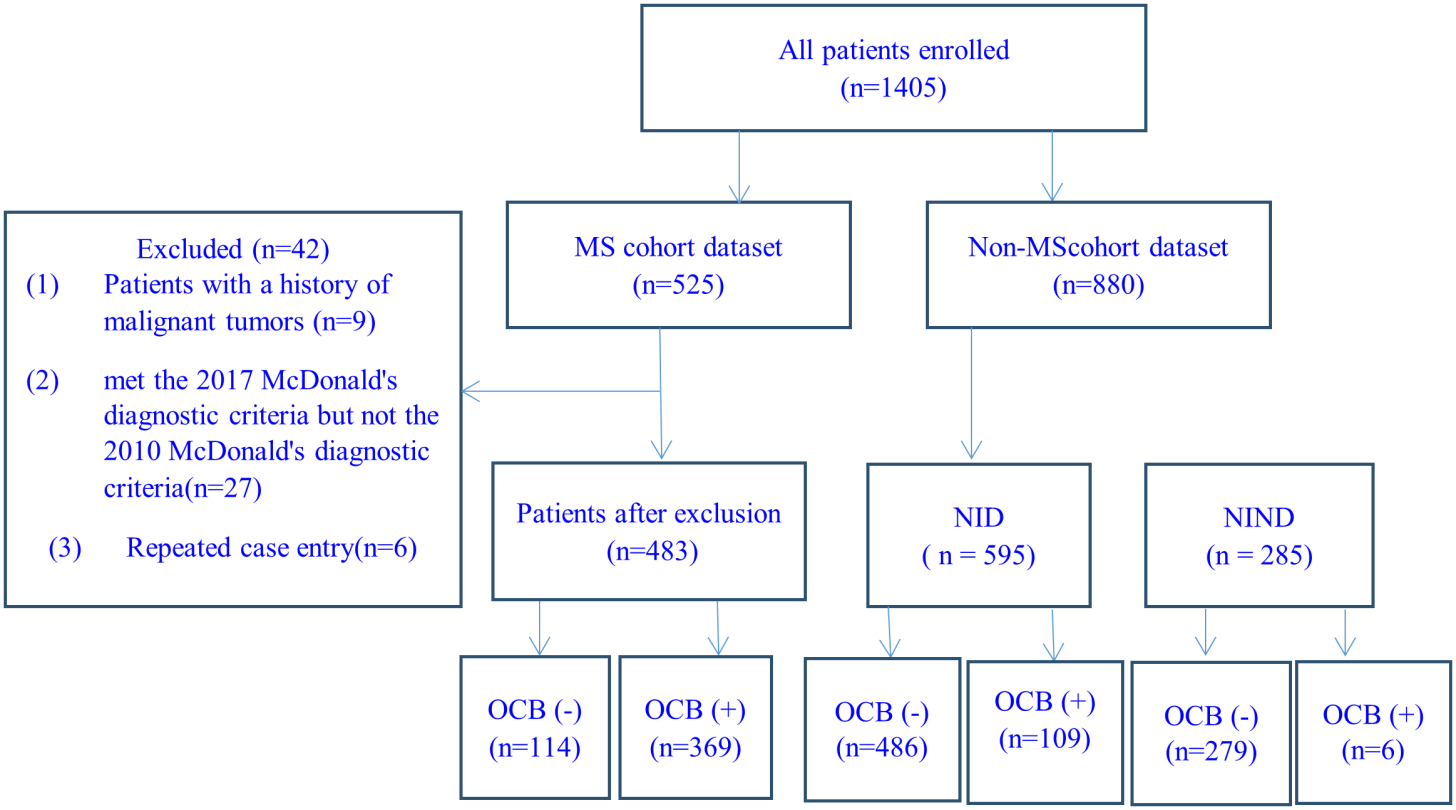
Case-screening flowchart. MS, multiple sclerosis; NID, neuro-inflammatory diseases; NIND: neurological non-inflammatory diseases.

In this study, the 2010 McDonald criteria (27) was required to be used for MS diagnosis, and a total of 525 MS patients were consecutively recruited and met the below inclusive criteria: (1) Chinese origin; (2) 14 to 65 years old; (3) two experienced neurologists in each center confirmed the diagnosis of MS independently from the collected CRF in reference to the original medical record when needed; (4) data on demography, CSF test, MRI examination, disease duration, numbers of relapse, annualized relapse rate (ARR), Expanded Disability Status Scale (EDSS), concomitant autoantibodies of or clinical diagnosis of other autoimmune diseases were available. 42 cases were excluded for the following reasons: 9 cases due to concurrent malignant tumors, 27 cases due to meeting the 2017 McDonald criteria but not the 2010 McDonald criteria, and 6 repeated-entry cases from different regional referring neurology centers. The remaining 483 patients were analyzed (**Figure 1**). The concomitant connective tissue disease (CTD) or autoimmune thyroid disease (AITD) was diagnosed according to relevant diagnostic criteria by consulting rheumatologists or endocrinologists. The number of attacks during the entire disease duration and the EDSS were recorded at the time of CSF collection.

CSF data of 880 consecutively recruited patients with the final diagnosis of other diseases in the 12 centers were collected during the same period, and used as the control group in the evaluation of the diagnostic performance of CSF-OCB in MS. All included patients were: (1) Chinese origin; (2) age of 14 to 65 years; (3) no MS history and two experienced neurologists excluded the diagnosis of MS when differential diagnosis presented; (4) data on demography and CSF-OCB were available. The 880 patients with non-MS diseases were divided into 2 categories: (1) neuro-inflammatory diseases (NID, n=595), including NMOSD (280 cases), MOGAD (45 cases), acute disseminated encephalomyelitis (ADEM) (15 cases), Guillain-Barré syndrome (127 cases) and autoimmune encephalitis (128 cases); (2)non-inflammatory neurological diseases (NIND, n=285), including primary headaches (56 cases), idiopathic epilepsy (20 cases), cerebrovascular diseases (79 cases), dementia (17 cases), motor neuron disease (23 cases), parkinsonism (6 cases), multiple system atrophy (6 cases), spinal vascular disease (7 cases), central nervous system involvement related to acute leukemia (10 cases), somatization disorder (7 cases), metabolic neuropathy (34 cases), hereditary neuropathy (14 cases), normal pressure hydrocephalus (4 cases) and peripheral vertigo (2 cases).

### Evaluation of the blood-CSF barrier and intrathecal IgG synthesis

The concentrations of IgG and albumin in serum and CSF were determined by the turbidimetric scattering method. The BCB permeability was assessed using CSF/serum albumin quotient: Q_Alb_ = CSF-Alb [mg/l]/serum-Alb [g/l]. Increased BCB permeability is defined as Q_Alb_>4+ (age/15). The IgG index, calculated as (CSF-IgG/serum-IgG)/(CSF-Alb/serum-Alb), is a measure of intrathecal IgG synthesis. An IgG index below 0.7 is considered normal. Tourtellotte IgG synthesis rate (IgG-SR), calculated as [(CSF IgG- serum IgG/369)- (CSF albumin- serum albumin/230) ×(serum IgG/serum albumin)]× 0.43 × 5, is an approach to determine intrathecal IgG synthesis with adjusting of BCB permeability. An IgG-SR below 3.3 mg/24 hours is considered normal (28, 29).

### OCB detection

According to the Isoelectric Focusing Electrophoresis (IEF) SOP for the detection of CSF-OCB (26, 30), the detection was performed with Sebia HYDRASYS 2 Isofocusing system PN1211 (France) according to the manufacturer’s instructions for the evaluation of IgG OCB. Briefly, CSF and serum samples were run in parallel at a concentration of 10-20 mg/L. After electrophoresis, the gel was incubated with peroxidase-labeled anti-IgG antibodies, and the bands were displayed using TTF1/2 chromogenic agents. Two inspectors independently interpreted the electrophoresis results according to the key points of interpretation, including the presence, number, and patterns of bands in the serum and CSF.

The electrophoresis results were classified into 5 main OCB types. Type I: no bands in both serum and CSF; Type II: ≥ 2 bands in CSF and no band in serum; Type III: additional bands in CSF despite of bands in serum; Type IV: identical bands in both serum and CSF; Type V: twin bands with regular and periodic spacing in both serum and CSF. CSF-OCB positivity was defined as either type II or III bands (29).

### Statistical analysis

Patient characters were presented as numbers (percentages), mean ± standard deviation (SD), or median (interquartile range, IQR). Pearson χ2 test and Fisher’s exact test, as well as Student’s T test or Mann-Whitney U test, were used for comparison between OCB-positive and OCB-negative groups.

The diagnostic performance of OCB positivity was evaluated as (15):

Accuracy: [(TP+TN)/(TP+TN+FP+FN)]
Sensitivity: [TP/(TP+FN)]
Specificity: [TN/(TN+FP)]
Positive Predictive Value (PPV): [TP/(TP+FP)]
Negative Predictive Value (NPV): [TN/(TN+FN)]
The likelihood ratio for positive test result (PLR): sensitivity/(1-specificity)
The likelihood ratio for negative test result (NLR): (1-sensitivity)/specificity

True positive (TP) was defined as MS according to McDonald 2010 criteria and positive OCB, true negative (TN) was defined as non-MS and negative OCB, false positive (FP) was defined as non-MS but positive OCB, and false negative (FN) was defined as MS but negative OCB.

A two-tailed *p* < 0.05 was considered statistically significant. All statistical analysis was performed using SPSS 26.0 software (IBM SPSS Statistics for Windows, Version 26.0. Armonk, NY: IBM Corp).

## Results

### General characteristics of patients with MS or other CNS diseases

The general characteristics of patients with MS were shown in **Table 1**. The mean age of 483 MS patients was 33.63 ± 10.58 years old. Females accounted for 63.2% of overall patients (female/male=2:1). 369 (76.4%) MS patients were OCB-positive (344 with type II, and 25 with type III), and 114 (23.6%) MS patients were OCB-negative (112 with type I, and 2 with type IV) (**Table 1**).

**Table 1:** The clinical characteristics of MS patients with different CSF-OCB.

Item	Overall	CSF-OCB		*p* value
		Negative	Positive	
Age (years), mean ± SD	33.63 ± 10.58	34.95 ± 11.55	33.23 ± 10.24	0.129
Age ≤ 50, n (%)	443	100 (87.7)	343 (93.0)	0.076
> 50, n (%)	40	14(12.3)	26(7.0)	
Sex, n (%)				
Male	178	48 (42.1)	130 (35.2)	0.184
Female	305	66 (57.9)	239 (64.8)	
With a history of other autoimmune disease				
No	452	99(86.8)	353(95.7)	0.001
Yes	31	15(13.2)	16(4.3)	
Time from symptom onset to diagnosis, months (P_25_, P_75_)	14.80 (2.13, 44.87)	23.70 (5.05, 50.73)	13.20 (1.58, 42.77)	0.020
Disease duration, n (%)				
≤1 year	218	47 (41.2)	171 (46.3)	0.499
1-5 years	173	46 (40.4)	127 (34.4)	
>5 years	92	21 (18.4)	71 (19.2)	
The number of attacks, mean #	2.45 ± 2.01	2.43 ± 2.15	2.48 ± 1.47	0.824
1-2 attacks	316	75 (65.8)	241(65.8)	0.784
3-5 attacks	144	33 (28.9)	111(30.3)	
>5 attacks	20	6 (5.3)	14 (3.8)	
ARR (P_25_, P_75_)	0.66 (0.31, 1.29)	0.66 (0.31, 1.00)	0.65 (0.30, 1.33)	0.907
EDSS, mean ± SD # #	2.54 ± 1.76	2.58 ± 1.79	2.42 ± 1.67	0.382
EDSS < 3, n (%)	309	70 (61.9)	239 (67.0)	0.224
3 ≤ EDSS ≤ 6, n (%)	140	40 (35.4)	100 (28.0)	
EDSS > 6, n (%)	21	3 (2.7)	18 (5.0)	

^#^Data missing in 3 patients; ^##^Data missing in 13 patients.

CSF, cerebrospinal fluid; OCB, oligoclonal bands; CTD, connective tissue disease; AITD, autoimmune thyroid disease; ARR, annualized relapse rate; EDSS, Expanded Disability Status Scale.

880 non-MS patients included 595 NID patients and 285 NIND patients. The mean age was 42.10 ± 16.71 years in NID patients and 45.53 ± 18.21 years in NIND patients. The female/male ratio was 1.2:1 in NID patients and 0.8:1 in NIND patients. 109 NID patients (18.3%) were OCB-positive (102 with type II, and 7 with type III), and 6 NIND patients (2.1%) were OCB-positive (5 with type II, and 1 with type III).

The patients with MS had higher prevalence of CSF-OCB than those with non-MS (76.4% vs 13.1%, P<0.001). In comparison with the NIND, NID patients had more percentage of positive CSF-OCB(18.3% vs 2.1%, P<0.001), but had less percentage of positive CSF-OCB when compared with MS (18.3% vs 76.4%, P<0.001).

### Diagnostic performance of CSF-OCB for differentiating MS from other CNS diseases

The diagnostic performance of CSF-OCB differentiating MS from non-MS patients yielded an accuracy of 83%, a sensitivity of 76%, a specificity of 87%, a PPV of 76%, a NPV of 87%, a PLR of 5.85, and a NLR of 0.27. The diagnostic performance in differentiating MS from NID patients showed 79%, 76%, 82%, 77%, 81%, 4.17, and 0.29, respectively. The diagnostic performance in differentiating MS from NIND patients showed 84%, 76%, 98%, 98%, 71%, 36.29, and 0.24, respectively (**Table 2**).

**Table 2:** Diagnostic value of CSF-OCB between MS and non-MS.

	Total, n	CSF-OCB		Accuracy (95% CI)	Sensitivity (95% CI)	Specificity (95% CI)	PPV (95% CI)	NPV (95% CI)	PLR (95% CI)	NLR (95% CI)
		Negative, n	Positive, n							
MS vs non-MS										
MS	483	114	369	0.83 (0.81, 0.85)	0.76 (0.72, 0.80)	0.87 (0.85, 0.89)	0.76 (0.72, 0.80)	0.87 (0.85, 0.89)	5.85 (4.90, 6.98)	0.27 (0.23, 0.32)
Non-MS	880	765	115							
MS vs NID										
MS	483	114	369	0.79 (0.77, 0.82	0.76 (0.72, 0.80)	0.82 (0.78, 0.85)	0.77 (0.73, 0.81)	0.81 (0.78, 0.84)	4.17 (3.49, 4.98)	0.29 (0.25, 0.34)
NID	595	486	109							
MS vs CIDD										
MS	483	114	369	0.77 (0.74, 0,80)	0.76 (0.72, 0.80)	0.79 (0.74, 0,84)	0.86 (0.83, 0.90)	0.66 (0.61, 0.71)	3.64 (2.78, 4.49)	0.30 (0.25, 0.35)
CIDD	276	218	58							
MS vs NIND										
MS	483	114	369	0.84 (0.82, 0.87)	0.76 (0.72, 0.80)	0.98 (0.95, 0.99)	0.98 (0.97, 0.99)	0.71 (0.66, 0.75)	36.29 (16.42, 80.22)	0.24 (0.21, 0.28)
NIND	285	279	6							

CNS, the central nervous system; MS, multiple sclerosis; non-MS, CNS disease other than MS; NID, CNS inflammatory diseases; CIDD, CNS inflammatory demyelinating diseases; NIND, CNS non-inflammatory diseases; PPV, Positive predictive value; NPV, Negative predictive value; PLR, Likelihood ratio for positive test results; NLR, Likelihood ratio for negative test results.

Moreover, considering that in most clinical cases, it is mainly CNS inflammatory demyelinating diseases (CIDD) that needs to be carefully distinguished from MS, we extracted data on the OCB status of the patients with CIDD (including NMOSD, MOGAD, ADEM) and compared it with MS patients, the diagnostic performance in differentiating MS from CIDD patients showed an accuracy of 77%, a sensitivity of 76%, a specificity of 79%, a PPV of 86%, a NPV of 66%, a PLR of 3.64, and a NLR of 0.30 (**Table 2**).

### Association between clinical characteristics and CSF-OCB positivity

**Figure 2:**
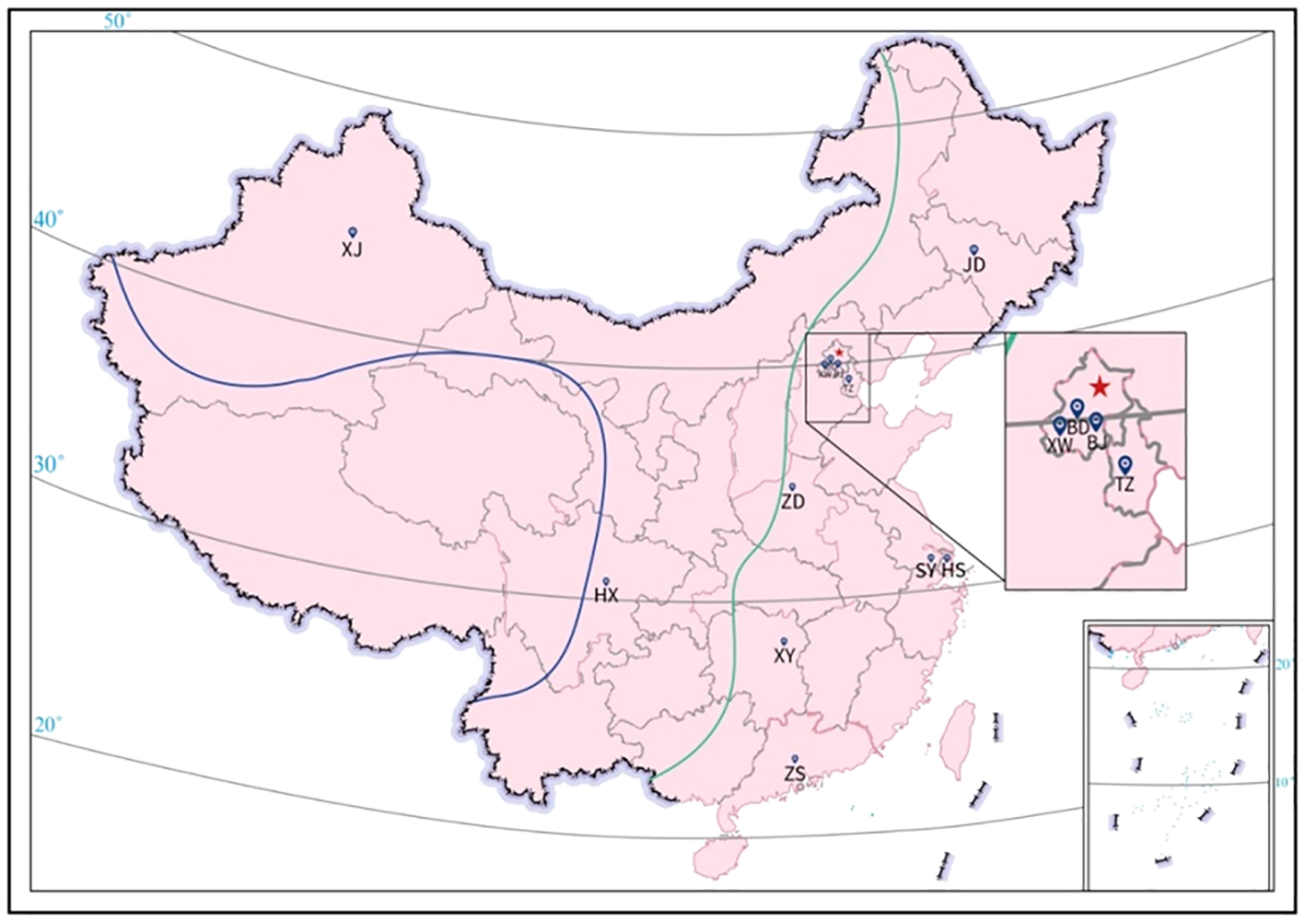
Geographical distribution of participating neurology centers. BD, Peking University First Hospital; BJ, Beijing Hospital; HS, Huashan Hospital affiliated to Fudan University; HX, West China Hospital of Sichuan University; JD, The First Hospital of Jilin University; SY, The First Affiliated Hospital of Soochow University; TZ; Tianjin Medical University General Hospital; XJ, People’s Hospital of Xinjiang Uygur Autonomous Region; XW, Xuanwu Hospital, Capital Medical University; XY, Xiangya Hospital of Central South University; ZD, The First Affiliated Hospital of Zhengzhou University; ZS, The Third Affiliated Hospital of Sun Yat-sen University. All patients were recruited from the 12 neurology centers in China and originated from 25 provinces and Beijing, Shanghai, Guangzhou, Chongqing (except Tibet, Hainan, Hong Kong, Macau, and Taiwan) spanning 20° and 50° North Latitude (20°-50°N). The altitude of China decreases from west to east and is commonly divided into 3 subregions: < 500m (east of green line), 1000-2000m (between green and blue line), and >4000m (southwest of blue line).

The included MS patients were from 25 provinces and 4 cities (Beijing, Shanghai, Guangzhou, and Chongqing) (**Figure 2**). Based on the characteristics of the geographical condition and human geography in China, they were divided into 7 regions, including Northeast (Heilongjiang, Jilin, and Liaoning), North (Beijing, Tianjin, Shanxi, Hebei, and Inner Mongolia), East (Shanghai, Jiangsu, Zhejiang, Anhui, Jiangxi, Shandong and Fujian), Central (Henan, Hunan, and Hubei), South (Guangdong and Guangxi), Southwest (Chongqing, Sichuan, Guizhou, and Yunnan), and Northwest (Shanxi, Gansu, Qinghai, Ningxia, and Xinjiang). The prevalence of CSF-OCB was around 60% in MS patients originating in the East and South regions and more than 80% in the other regions. The provinces and cities were also classified into low latitude (20°-30°N), middle latitude (31°-40°N), and high latitude (41°-50°N) regions (**Figure 2**). The prevalence of CSF-OCB in MS patients in these regions was 68.1% (81/119), 77.0% (217/282), and 86.6% (71/82), significantly higher in high-latitude regions (P=0.016) (**Table 3**). They were also classified into low-altitude (<500m) and high-altitude (>1000m) regions (**Figure 2**). The prevalence of CSF-OCB in MS patients at high altitudes (82.63%, 138/167) was significantly higher than those at low altitudes (73.1%, 231/316) (P=0.025) (**Table 3**).

**Table 3:** Geographical distribution of CSF-OCB positivity.

Geographical regions	Overall, n	CSF-OCB, n (%)		*p* value
		Negative	Positive	
Latitude				
Low (20° – 30°N)	119	38 (31.9)	81 (68.1)	0.016
Medium (31° – 40°N)	282	65 (23.0)	217 (77.0)	
High (41° – 50°N)	82	11 (13.4)	71 (86.6)	
Altitude				
Low (< 500 m)	316	85 (26.9)	231(73.1)	0.025
High (> 1000 m)	167	29 (17.4)	138 (82.6)	

As shown in **Table 1**, there were no differences about age and gender between OCB positive and negative patients (P=0.129, P=0.184). Time from symptom onset to diagnosis was significantly shorter in OCB-positive patients than that in OCB-negative patients (13.2 months vs. 23.7 months, P=0.020). There were fewer patients with concomitant autoimmune diseases in the OCB-positive patients (4.3%, 16/369) than in the OCB-negative patients (13.2%, 15/114) (P=0.001). No significant differences were observed among OCB status and disease duration, the number of attacks, ARR and EDSS (P=0.499, P=0.824, P=0.907, P=0.382) (**Table 1**).

### The association between CSF-OCB and MRI features

The attacks of MS affect various parts of the CNS, in this study T2 weighted MRI lesions in different locations of CNS were compared between OCB-positive and negative patients. More OCB-positive patients have periventricular lesions than OCB-negative patients (93.6% vs 86.5%, P= 0.017) (**Table 4**).

**Table 4:** The CSF-OCB and MRI lesions of MS patients.

Lesion distribution	Overall, n	CSF-OCB, n (%)		*p* value
		Negative	Positive	
Optic nerve				
**No**	435	104 (95.4)	331 (93.0)	0.365
**Yes**	30	5 (4.6)	25 (7.0)	
Cortical/Juxtacortical				
**No**	112	29 (26.1)	83 (23.3)	0.545
**Yes**	355	82 (73.9)	273 (76.7)	
Periventricular				
**No**	38	15 (13.5)	23 (6.4)	0.017
**Yes**	431	96 (86.5)	335 (93.6)	
Brain stem				
**No**	226	55 (49.5)	171 (47.8)	0.742
**Yes**	243	56 (50.4)	187 (52.2)	
Cerebellum				
**No**	355	84 (75.7)	271 (75.7)	0.996
**Yes**	114	27 (24.3)	87 (24.3)	
Cervical spinal cord				
**No**	170	45 (42.5)	125 (36.1)	0.239
**Yes**	282	61 (57.5)	221 (63.9)	
Thoracic spinal cord				
**No**	259	62 (59.0)	197 (57.9)	0.841
**Yes**	186	43 (41.0)	143 (42.1)	
Lumbar spinal cord				
**No**	408	92 (96.8)	316 (96.3)	0.816
**Yes**	15	3 (3.2)	12 (3.7)	

### The association between CSF-OCB and CSF parameters

The mean level of CSF protein was lower in OCB-positive patients than that in OCB-negative patients (340.00 mg/L vs 370.00 mg/L, P<0.001). Less OCB-positive MS patients had elevated Q_Alb_ in comparison with OCB-negative MS patients (16.0%, 37/231 vs 29.3%, 22/75, P=0.011). The proportions of MS patients with increased IgG index and abnormal IgG-SR were greater in OCB-positive patients than those in OCB-negative patients (69.8%, 215/308 vs 33.7%, 32/95, P<0.001; 69.3%, 212/306 vs 38.5%, 37/96, P<0.001, respectively).

## Discussion

In our study, the enrolled MS patients originated from 25 provinces and 4 cities, covering most regions in mainland of China, which depicted a representative nationwide portrait of Chinese MS patients, and provided insight into the clinical and diagnostic value of CSF-OCB in Chinese MS patients. As we know, this is the first report about nationwide data on CSF-OCB positivity and its diagnostic performance in Chinese MS patients, especially in the virtue of common SOP.

We adopted the 2010 McDonald Criteria because it did not need the information of CSF-OCB, and had fairly good sensitivity for diagnosing MS in CIS patients (27). Moreover, the prevalence of CSF-OCB is easily compared with earlier researchers. Importantly, this would avoid bias from the incorporation of OCB in diagnostic studies because the 2017 McDonald criteria and other diagnostic criteria all incorporate OCB as supporting conditions (15).

OCB suggests intrathecally synthesized immunoglobulin in response to B cell activation as a result of CNS inflammation (12). It is not only detected in MS but also in other neurological disease (19). Several previous reports had shown that CSF-OCB had a high specificity for diagnosing MS. In these articles, MS was often compared to healthy individuals or patients with non-neurological inflammatory diseases (12, 30–32). However, A meta-analysis had shown that when patients with neurological inflammatory diseases were used as the control group, the specificity of CSF-OCB for diagnosing MS would reduce from 94% to 61% (32). These suggested that the differences in the control group used in research could greatly affect the evaluation of the specificity of CSF-OCB in diagnosing MS, and was one of the reasons for the significant differences about specificity reported in various studies. In clinical practice, the differential diagnosis between MS and neurological inflammatory diseases is crucial. In this study, 18.3% of patients in the NID were CSF-OCB positive, which is significantly higher than those in the NIND. Therefore, we included NID and NIND patients as the controls, and found that when NIND was used as the control, the specificity of CSF-OCB for diagnosing MS could reach 98%, while when NID was used as the control, the specificity dropped to 77%.

In clinical practice, it is crucial for MS to be distinguished from CIDD. The significant difference in OCB prevalence between MS and CIDD (76.4% vs 21.0%) shown in this study (diagnostic spectivity 79%) suggests that negative CSF-OCB could be a “red flag” for the diagnosis of MS. When some patients with central demyelinating disease have clinical and/or imaging manifestations that are very similar to MS, and may even be diagnosed as MS according to MS diagnostic criteria at a certain time point, their negative OCB results need to be constantly reminded to doctors during long-term follow-up to pay attention to whether changes in the patient’s clinical and imaging manifestations that were inconsistent with MS, so as to timely revise the diagnosis. Moreover, the time from the onset of disease to clinical diagnosis in our cohort was almost one-year earlier in OCB-positive patients than that in OCB-negative patients, supporting that OCB detection also facilitates early MS diagnosis for Chinese patients with MS.

In previous studies, Zheng et al. reported that the OCB positive rate was 62.5% in Zhejiang, a province of East China (17). Lu et al. have revealed that the OCB positive rate was 59.8% in Guangdong Province and Hong Kong SAR, two regions belonging to South China (20). In the survey of MS patients in north China, GU et al. and Chen et al. reported that the positive rates of OCB were 69.3% and 72.7% respectively (33, 34). According to China’s seven commonly used geographical divisions, China’s territory can be divided into North, South, Central, East, Northeast, Northwest, and Southwest. These 7 regions have obvious differences in topography, climate, vegetation types, production modes, habits, customs, and culture. In this study, the overall OCB positivity was 76.4% in Chinese patients with MS, and was about 60% in East and South China which was consistent with the results reported by Zheng et al. and Lu et al (17, 20), and was about 90% in North China, slightly higher than the results reported by GU et al. and Chen et al (33, 34). Thus, the inconsistencies of the previous reports were well explained in the analysis considering the geographical distribution difference of MS patients as the dominant factor.

Our results also showed that the geographical distribution of high OCB prevalence demonstrated a high-latitude feature. Lechner-Scott et al. have prospectively collected and analyzed CSF-OCB data from MSBase, a large international, multi-center database, and found that the frequency of OCB increased with latitude (35). In Lechner-Scott’s paper, the OCB positive rate was 85~100% in the regions located North of 40°N, which is similar to the latitudes in our study. The OCB positive rate was 50~90% in the 30°~40°N regions, and again, this is similar to the latitudes in this study. As to the regions in 20°~30°N, the OCB positive was 35% and 59%, which is lower than the same latitude in our investigation. In another report on the positive OCB and/or increased IgG index and latitude from Japan (36), CSF-OCB prevalence of MS in the northern region(42°-45°N) was higher than that in the southern region (33°-35°N), the average positive rate of the OCB and/or increased IgG index was 58.7%, and was similar to that reported in this study in the eastern coastal region of China (61.5%), which geographical characteristics are similar to those of Japan on the whole. Latitudinally the OCB prevalence in China was comparable to Europe and the United States (18, 35, 37), especially in high-latitudinal regions.

As to the altitude, only a few papers have revealed the relationship between MS morbidity and altitude (38, 39), but no publications had reported the relationship between CSF-OCB positivity and altitude. As shown in **Table 3**, we noticed that the OCB prevalence of patients observed in this study showed a trend related to altitude, that was, the OCB prevalence of patients in high-altitude areas was higher than that in low-altitude areas. As we know, this is the first report about the relationship between CSF-OCB prevalence and altitude.

People living in high-latitude and high-altitude areas of China, as a whole, tend to have a diet dominated by rice and pasta, with less consumption of vegetables, fruits, and fish, and have lower levels of vitamin D (40, 41). They also have specific human leukocyte antigen (HLA) enabling them to better adapt to high-altitude and high-latitude environments (42, 43).It has been confirmed that the risk of MS was associated with various geographic, environmental, ethnic, and lifestyle factors, especially genetic susceptibility(HLA), diet customs and vitamin D levels, which might promote the occurrence of MS by the influence on the status or function of immune cells, and logically the state of OCB produced by plasma cells in CNS (36, 44–48). However, the relationship between these factors and the productivity of OCB in China still lacks strong validation, and further research is needed to verify.

There were many studies on the correlation between OCB positivity and long-term MS activity and prognosis, but the results were controversial (49). Some studies reported that OCB positivity correlated with disease activity, such as ARR, and EDSS (50, 51), but in some other studies, the opposite conclusion was reported (20, 52). In this study, no significant differences were observed among OCB status and EDSS, ARR, disease course, and the number of relapses. Because this study was only a cross-sectional observation study, the correlation between OCB positivity and long-term MS activity and prognosis in Chinese people needs further study.

Both MRI and CSF-OCB are key indicators in the diagnostic criteria of MS (15). In this study, the lesion locations of MS were summarized as optical nerve, cortical/juxtacortical, periventricular, brain stem, cerebellum, cervical spinal cord, thoracic spinal cord, and lumbar spinal cord. Zhao et al. reported that in comparison with the OCB-negative MS patients, the OCB-positive group had a higher proportion of cerebellar lesions (53); Huttner et al. Suggested that MRI lesions and OCB status were independent of each other (54). However, we found that patients with positive OCB had more periventricular lesions than patients with negative OCB, while there was no significant difference in the proportion of patients with other lesions.

The BCB is an important physiological natural barrier of the CNS and peripheral environment, which can prevent the entry of various proteins and chemicals, including immunoglobulin. The presence of OCB in CSF indicates the existence of chronic persistent inflammation in CNS, suggesting the production of immunoglobulin in CNS, rather than that caused by the peripheral immunoglobulin entering CNS (55). In this study, BCB disruption was only observed in a small proportion (~20%) of MS patients in which most of the patients were OCB negative, and was negatively correlated to OCB positivity, as with previous studies (12, 56, 57).

IgG index and IgG-SR are important parameters of CSF analysis about CNS immune response. Like OCB, they are also designed to evaluate the presence of immunoglobulin synthesis and inflammatory processes in CNS and are widely used in the clinical diagnosis of MS (10, 29). Several studies have reported that an IgG index>0.7 was closely correlated with OCB positivity and was a prognostic marker of early disease activity (58, 59).In agreement with these reports, this study also showed that the proportions of MS patients with increased IgG index and abnormal IgG-SR were greater in OCB-positive patients than those in OCB-negative patients (28, 58–60).

The strength of this study is based on the following: 1. The sample size is large and the SOP adopted in CSF-OCB testing was previously validated. 2. The twelve regional referring MS centers cover the difference in latitude and altitude and represent the typical culture and convention in China. 3. Accurate diagnosis of MS and non-MS demyelinating diseases was guaranteed with the same CRF and we aimed to evaluate the diagnostic performance of CSF-OCB in MS patients, the possibility of misclassification of MS and non-MS demyelinating diseases was tiny based on the consensus from regional and nationwide experts on doubtful patients. 4. Using the 2010 Mcdonald criteria, which is based only with clinical and radiological data, avoiding the inclusion bias by using MS patients diagnosed with criteria that adopt the role of CSF-OCB.

There were some limitations in this study: 1. Only cross-sectional EDSS was collected, and the relapse risk was analyzed with retrospective data. 2. Long-term changes in CSF-OCB status in MS patients and non-MS demyelinating disease patients were not addressed. 3. The association between CSF-OCB status and some radiological features of MS could not be analyzed due to the cross-sectional design and only some retrospective data were acquired by reviewing the case records. Nevertheless, these are not the main focus of this study. We are collecting relevant data in a prospective cohort among all participating centers.

In conclusion, this study reported, for the first time, that the nationwide prevalence of CSF-OCB was 76.4% and conducive to early diagnosis in Chinese patients with MS, and demonstrated a good performance in differentiating MS from other CNS diseases, suggesting that CSF-OCB is a valuable clinical indicator for the diagnosis of MS in Chinese patients. In addition, the CSF-OCB prevalence showed a correlation with high latitude and altitude, reflecting the characteristics of regional distribution of OCB prevalence in Chinese patients with MS.

## Data availability statement

The original contributions presented in the study are included in the article/supplementary material. Further inquiries can be directed to the corresponding authors.

## Ethics statement

The studies involving humans were approved by the Ethics Committee of Shanghai Huashan Hospital. The studies were conducted in accordance with the local legislation and institutional requirements. The participants provided their written informed consent to participate in this study.

## Author contributions

XZ: Conceptualization, Data curation, Formal Analysis, Investigation, Methodology, Software, Visualization, Writing – original draft, Writing – review & editing. HH: Conceptualization, Data curation, Investigation, Methodology, Writing – original draft. TJ: Data curation, Investigation, Methodology, Writing – review & editing. WQ: Conceptualization, Data curation, Investigation, Methodology, Writing – review & editing. HuY: Conceptualization, Data curation, Investigation, Methodology, Writing – review & editing. QX: Conceptualization, Data curation, Investigation, Methodology, Writing – review & editing. JYi: Conceptualization, Data curation, Investigation, Writing – review & editing. ZS: Data curation, Investigation, Methodology, Writing – review & editing. HaY: Conceptualization, Data curation, Investigation, Writing – review & editing. XJ: Data curation, Investigation, Writing – review & editing. XS: Data curation, Investigation, Methodology, Writing – review & editing. QZ: Data curation, Investigation, Methodology, Writing – review & editing. XL: Data curation, Investigation, Methodology, Writing – original draft. JW: Writing – review & editing, Data curation, Investigation, Methodology. HuL: Data curation, Investigation, Software, Writing – review & editing. XH: Data curation, Investigation, Software, Writing – review & editing. JYa: Data curation, Investigation, Software, Writing – review & editing. YL: Data curation, Methodology, Writing – review & editing. SL: Data curation, Investigation, Methodology, Software, Writing – review & editing. AL: Conceptualization, Data curation, Investigation, Methodology, Writing – review & editing. FG: Conceptualization, Data curation, Investigation, Writing – review & editing. SH: Data curation, Methodology, Software, Writing – review & editing. SC: Conceptualization, Investigation, Methodology, Writing – review & editing. DD: Conceptualization, Investigation, Methodology, Software, Writing – review & editing. HZ: Conceptualization, Data curation, Investigation, Supervision, Visualization, Writing – review & editing. HaL: Conceptualization, Data curation, Investigation, Supervision, Visualization, Writing – review & editing. XC: Conceptualization, Data curation, Funding acquisition, Investigation, Methodology, Resources, Supervision, Validation, Visualization, Writing – review & editing.

## Appendix 1 The information regarding MRI collected in CRF.

**Table:**

MRI with and without intravenous gadolinium-based contrast agent administration	
Lesion distribution:	
Optic nerve: Yes/No Cortical/Juxtacortical: Yes/No Periventricular: Yes/No Brain stem: Yes/No Cerebellum: Yes/No Cervical spinal cord: Yes/No Thoracic spinal cord: Yes/No Lumbar spinal cord: Yes/No	Contrast-enhancing lesions: Yes/No Contrast-enhancing lesions: Yes/No Contrast-enhancing lesions: Yes/No Contrast-enhancing lesions: Yes/No Contrast-enhancing lesions: Yes/No Contrast-enhancing lesions: Yes/No Contrast-enhancing lesions: Yes/No Contrast-enhancing lesions: Yes/No
Are the radiological McDonald criteria fulfilled:	
• Dissemination in time: Yes/No • Dissemination in space: Yes/No	

Standardized imaging protocol: ①At least 1·5T; 3T if available; ②Core sequences: axial T2-weighted, and T1-weighted with and without gadolinium;T2-weighted fluid-attenuated inversion recovery.
